# Physics-Informed and Explainable Artificial Intelligence for Nanomaterial-Based Biosensors: From Sensor Design and Signal Processing to Clinical Translation

**DOI:** 10.3390/bioengineering13101133

**Published:** 2026-09-28

**Authors:** Stefano Bellucci

**Affiliations:** 1 Qi S.r.l., Via Monte d’Oro 2/A, Pomezia, 00071 Roma, Italy; stefano.bellucci@lnf.infn.it; 2 Universidad Ecotec, Km. 13.5 Samborondón, Samborondón 092302, Ecuador

**Keywords:** artificial intelligence, machine learning, explainable AI, physics-informed machine learning, biosensors, graphene, two-dimensional materials, surface plasmon resonance, terahertz metasurfaces, electrochemical biosensors

## Abstract

Artificial intelligence (AI) is increasingly used in biosensing, yet its role is often limited to post-processing or high-accuracy regression on simulation-generated datasets. This critical narrative review examines physics-informed and explainable AI for nanomaterial-based biosensors, emphasizing graphene and other two-dimensional materials, surface plasmon resonance (SPR), terahertz (THz) metasurfaces, electrochemical platforms, field-effect transistors, surface-enhanced Raman spectroscopy, and wearable systems. Recent peer-reviewed studies and relevant technical guidance were critically compared with respect to sensing physics, data provenance, AI task, interpretability, validation strategy, and evidence level. Across these modalities, AI supports forward surrogate modeling, inverse design, spectral interpretation, classification, calibration, and uncertainty-aware decision support, but predictive accuracy alone does not establish translational maturity. Particular attention is given to explainable AI, the distinction between physics-guided and genuinely physics-informed learning, small-data validation, fabrication tolerance, drift, and the simulation-to-experiment gap. Quantitative comparisons place reported SPR and THz sensitivities in context, while independent experimental studies provide benchmarks for real-device and biomedical validation. Clinically credible intelligent biosensors should combine mechanistic constraints, uncertainty quantification, device- and batch-aware validation, interpretable features, realistic biological matrices, and prospective evaluation.

## 1. Introduction

Artificial intelligence (AI) and bioengineering are converging at several levels, from biological data analysis and imaging to biomaterials, digital twins, intelligent biosensors and personalized medicine. Giansanti has recently framed this convergence as a transition from the use of isolated machine-learning tools toward integrated intelligent bioengineering systems [1]. Biosensors are a particularly suitable test bed for this transition because they sit at the interface between materials, physics, chemistry, biology, electronics and decision-making. Their output is generated by a physical transduction process, but the measured signal is also affected by noise, device variability, matrix effects, drift, and the structure of the downstream inference task.

Nanomaterials enlarge this design space. Graphene and related two-dimensional (2D) materials can modify surface adsorption, optical field confinement, charge transfer and electronic transport, while plasmonic metals, oxides, semiconductors and hybrid heterostructures provide additional degrees of freedom in thickness, composition and geometry. In SPR biosensors, graphene can improve surface interaction and sensitivity, but practical performance remains strongly dependent on optical configuration, losses, integration quality, and the biological recognition layer [2]. Graphene/Si3N4 architectures for SARS-CoV-2 sensing further illustrate that the highest sensitivity and the highest robustness or figure of merit do not necessarily occur in the same multilayer configuration [3].

The same multidimensionality that makes nanomaterial biosensors powerful also makes brute-force optimization expensive and interpretation difficult. Machine learning (ML) can serve as a surrogate for finite-element, transfer-matrix, electrochemical, or kinetic models; it can classify complex sensor patterns; and it can help solve inverse-design problems. In electrochemical sensing, recent reviews already document the value of ML for noisy signals, interference, electrode fouling, high-throughput measurements, and continuous monitoring [4]. Yet a central problem remains: a model can produce an excellent R^2^ or classification accuracy while learning correlations that are physically fragile, device-specific, or caused by leakage between training and test data.

The central question addressed here is narrower than general ‘AI in biosensors’: how can sensor physics, explainability, and uncertainty be integrated so that AI improves both performance and scientific credibility? Nanomaterial-based optical and plasmonic biosensors provide a useful test case because they are governed by well-defined electromagnetic relationships and are increasingly combined with ML/XAI methods. The same framework also applies to electrochemical, impedance, and field-effect biosensors. The overall workflow is summarized in Figure 1.

Recent literature shows a clear progression from nanomaterial-enabled transduction to AI-enabled inference. Carbon-nanomaterial screen-printed electrochemical platforms have been experimentally established to show that material chemistry and interfacial electron transfer can produce large changes in analytical sensitivity [5]. Independent reviews and primary studies now extend this progression to plasmonic, electrochemical, SERS, GFET and wearable platforms, where machine learning is used for surrogate modeling, feature attribution, classification, calibration transfer and uncertainty-aware analysis [2,6,7,8,9,10,11,12,13,14,15,16,17,18,19,20,21]. These studies help separate improvements arising from transducer physics from those introduced by the learning algorithm.

The wider literature shows the same evolution. Experimental LSPR imaging combined with machine learning has already demonstrated direct SARS-CoV-2 particle detection [22], while recent SERS studies combine measured spectra with supervised learning and Bayesian optimization [23]. Broader reviews cover graphene electrochemical biosensors [24], machine-learning-assisted 2D optical biosensors [6], and PCF-SPR modeling with ML [25]. These studies are particularly useful for evaluating how conclusions drawn from simulation-based optimization compare with results obtained from measured signals.

## 2. Scope and Review Approach

This article is a critical narrative review rather than a formal systematic or scoping review. Representative peer-reviewed literature and relevant regulatory or technical guidance were identified through targeted literature searches, citation chaining, and verification against publisher or agency records. The discussion was organized around four connected areas: (i) graphene and two-dimensional-material biosensor physics; (ii) machine-learning-assisted sensor modeling and optimization; (iii) explainable artificial intelligence (XAI) for sensor design and signal interpretation; and (iv) physics-informed machine learning and translational requirements. The literature was updated through August 2026, with particular attention to studies published from 2023 to 2026. Recent reviews were used to map the main sensing modalities, while original studies using fabricated devices, measured spectra, wearable data, or patient-derived samples were prioritized where available. Foundational references on explainability, physics-informed learning, and clinical AI were retained to define the methodological framework.

The scope differs from recent broad reviews. Geddis et al. surveyed AI/ML across plasmonic and surface-enhanced sensing modalities [7], Din et al. reviewed the wider convergence of AI with 2D materials in biosensing and nanoelectronics [26], and Xu et al. addressed 2D materials for integrated sensing and information encoding [27]. Here the emphasis is methodological: how physics constraints, explainability, uncertainty, and evidence quality can be combined in a trustworthy design-to-translation workflow for nanomaterial biosensors.

Four terms are used consistently throughout this review. “ML-assisted” denotes a data-driven predictor, classifier, or optimizer. “Explainable ML” adds an attribution or interpretable-model layer such as SHAP or LIME. “Physics-guided ML” denotes models that use simulation-generated training data, physics-derived inputs, parameter bounds, priors, or post hoc physical screening, but do not enforce governing equations during learning. “Physics-informed AI” is reserved here for models in which a governing equation, conservation law, boundary condition, or differentiable mechanistic forward model enters the loss function, architecture, or inference procedure itself. Consequently, a neural network, GPR, random forest, or boosting model trained only on TMM/FEM grids is described as a simulation-trained surrogate or physics-guided model, not as physics-informed.

The literature considered spans SPR and plasmonic sensing, SERS, graphene field-effect transistors (GFETs), electrochemical and wearable biosensors, microfluidic systems, and THz platforms. Recent modality-specific reviews identify common challenges across these areas: information-rich but device-dependent signals, limited or heterogeneous datasets, batch and site effects, calibration transfer, and the need to match the learning task to the underlying measurement physics [2,28,29,30,31,32,33,34,35,36,37,38,39,40,41,42,43,44,45]. These recurring issues provide a common basis for comparing data provenance, validation design, and evidence quality across otherwise different sensing technologies.

During preparation and revision of this manuscript, AI tools were used for language-level drafting and structural editing in Section 2, Section 6, Section 7, Section 10, Section 11 and Section 12; for organizing comparative-table layouts; and for preparing the initial schematic layouts of selected figures. It was not used to select studies autonomously, determine evidence levels, generate or alter research data, calculate reported numerical results, or make the scientific conclusions. The author independently verified every citation, numerical value, interpretation, equation, table entry, and figure label against the cited sources, substantially revised the generated wording, and takes full responsibility for the final content.

## 3. Why Nanomaterial-Based Biosensors Are a Natural Target for AI

### 3.1. A High-Dimensional but Structured Design Space

A nanomaterial biosensor rarely has a single tunable parameter. Relevant variables may include metal thickness, dielectric thickness, number of graphene layers, 2D-material orientation, surface coverage, functionalization density, channel geometry, wavelength, incidence angle, electrolyte composition, receptor affinity, and analyte refractive index or concentration. Exhaustive parametric sweeps scale poorly as variables accumulate. ML surrogates can compress the mapping from this high-dimensional input space to sensor outputs such as resonance angle, wavelength shift, sensitivity, full width at half maximum (FWHM), figure of merit (FOM), impedance features, or classification probability.

Crucially, however, the space is structured by physics. In an SPR device, the resonance condition follows from electromagnetic coupling at a metal–dielectric interface; changes in thickness and complex permittivity alter phase matching and damping. In electrochemical sensors, transport, reaction kinetics, double-layer capacitance, and electrode surface state constrain the time- or frequency-domain response. In FET biosensors, electrostatics, carrier density, contact resistance, and screening determine the transduction. AI is therefore most useful when it exploits this structure instead of treating every feature as an arbitrary tabular variable.

### 3.2. Graphene and 2D Materials: Performance Gains and Trade-Offs

Graphene is attractive for biosensing because of its high surface area, electronic properties, optical absorption, mechanical strength, and capacity for surface functionalization. In graphene-enhanced SPR sensors, its role is not reducible to a generic “sensitivity enhancer”; depending on the optical stack, additional graphene can improve adsorption and field interaction while also modifying losses and resonance linewidth. Theoretical analysis of Au-, Ag- and bimetallic graphene SPR configurations showed that substrate selection, layer number and metal thickness affect sensitivity, detection accuracy and quality factor in different ways [46]. Broader independent reviews of SPR biosensing likewise emphasize the heterogeneity of materials, optical configurations and validation strategies and the need to interpret sensitivity together with linewidth, robustness and experimental evidence [2,7].

This trade-off structure is precisely where AI can add value. Instead of optimizing a single scalar target, multi-objective models can search for solutions that balance sensitivity, linewidth, FOM, fabrication tolerance and stability. For black phosphorus (BP), MXenes, transition-metal dichalcogenides (TMDs) and hybrid metal/2D stacks, AI can additionally help navigate anisotropy, oxidation susceptibility, interlayer coupling and large combinatorial material spaces. The objective should be a Pareto-optimal and fabrication-feasible design rather than a numerically maximal sensitivity obtained under idealized assumptions.

### 3.3. From Nanocarbon Transduction to AI-Enabled Resonant Biosensing

Several studies spanning electrochemical, SPR, and THz sensing illustrate which problems genuinely benefit from AI. In the 2022 uric-acid work, carbon-nanomaterial-modified screen-printed electrodes were characterized experimentally before being converted into an enzymatic amperometric biosensor [5]. The CO_2_H-MWNT platform reached a reported sensitivity of 418 μA μM^−1^ cm^−2^ and a detection limit of 0.5 nM, compared with 33 μA μM^−1^ cm^−2^ and 280 nM for the bare-electrode biosensor. These gains were obtained without machine learning: material functionalization, electron-transfer kinetics, and electrode architecture were the dominant variables. This provides a useful reference point for cases in which mechanistic materials optimization already explains the observed performance.

The optical design space becomes substantially more multidimensional when graphene layer number, metal ordering, metal thickness, dielectric loading, and additional 2D materials are varied simultaneously [46]. Numerical architectures incorporating bilayer MoS_2_ [47], MoSe_2_ [48] and graphene/Si_3_N_4_ [3] provide examples of physically interpretable parameter spaces for sensitivity analysis. Independent machine-learning studies of 2D-material SPR and PCF-SPR systems further show how such spaces can be compressed into fast surrogate models or searched through constrained optimization [8,9,10,49].

Recent independent studies add explicit machine-learning and explainability layers to optical-sensor design. PCF-SPR work has combined multiple regressors with SHAP-based feature attribution and, more recently, uncertainty quantification [8,49]. Fiber-optic SPR studies have used GPR together with SHAP and LIME to connect learned feature importance to known optical behavior [9], while multilayer SPR studies have coupled regression, SHAP analysis, finite-element checks and fabrication-tolerance perturbations [50]. These examples make the central methodological point: high predictive accuracy on a simulator-generated response surface is useful for engineering interpolation, but it is not equivalent to independent biomedical validation.

## 4. Where AI Enters the Biosensor Lifecycle

The main AI functions across the nanomaterial-biosensor lifecycle are summarized in Table 1.

### 4.1. Forward Surrogate Modeling

The most mature use of ML in optical biosensor design is surrogate modeling. A physics solver first generates a dataset by sweeping geometric, material, and analyte parameters. A regression model is then trained to reproduce quantities such as effective refractive index, confinement loss, resonance angle, or FOM. Once trained, evaluation is orders of magnitude faster than repeated full-wave or transfer-matrix simulations, enabling sensitivity analysis and optimization over a larger parameter space.

The value of this approach is illustrated by recent PCF-SPR studies. Khatun and Islam combined multiple regressors with SHAP explanations to predict optical properties and identify dominant design variables [8]. Their later ML/XAI framework added uncertainty quantification for effective index and confinement loss [49]. These studies represent a useful progression: predictive speed is combined with interpretability and confidence estimates rather than reported as a single high R^2^ value.

### 4.2. Inverse Design and Optimization

Forward prediction answers “what will this design do?” Inverse design asks the more useful engineering question: “which design produces the desired response?” Because different multilayer stacks can yield similar resonance metrics, inverse design is generally non-unique. A robust workflow therefore couples a fast surrogate with constraints on material availability, thickness ranges, oxidation stability, deposition resolution, and acceptable optical loss. Multi-objective optimization should return a set of feasible designs rather than a single numerical optimum. Recent work is also extending this concept toward ML-assisted inverse design of prism-based SPR sensors with SHAP-informed robustness analysis [51].

Physics-based surrogate modeling is also increasingly combined with SHAP, robustness testing, and optimization for multilayer SPR structures. A recent 2026 arsenic-sensing study generated a transfer-matrix dataset, compared regression models, used SHAP to identify dominant Cu and Ni thickness effects, validated the optical model by finite-element calculations, and assessed fabrication uncertainty by Monte Carlo perturbations [50]. This combination is closer to an engineering-grade AI workflow because it treats interpretability and manufacturing variability as part of the design problem.

### 4.3. Signal Processing, Classification and Continuous Monitoring

In experimental biosensing, AI operates on signals rather than ideal design parameters. Electrochemical and impedance sensors may generate multivariate curves whose useful information is distributed across peak shape, phase, frequency, and time. Optical devices may exhibit asymmetric or drifting resonances that make fixed fitting rules unreliable. ML can improve dip localization, baseline correction, denoising, multianalyte classification, and mapping from a high-dimensional response to analyte concentration. In continuous monitoring, algorithms can also learn subject- or device-specific baselines and identify drift.

This is where train/test design becomes decisive. Randomly splitting individual measurements can place nearly identical repeats from the same device, batch, sample, or simulation sweep in both training and test sets. The resulting score estimates interpolation within a familiar device rather than generalization to a new device or patient. Whenever the intended use involves unseen devices, batches, or subjects, the test split should occur at that level. The progression from prediction to experimentally validated use is illustrated in Figure 2.

Experimental AI workflows are more demanding because the model must cope with nuisance variation that is absent from ideal parameter sweeps. Liang et al. combined LSPR sensing, optical imaging, and ML for direct SARS-CoV-2 particle detection, reporting a 100 vp/mL limit of detection, >97% positive/negative classification accuracy, and R^2^ > 0.95 for concentration prediction [22]. In a different spectral regime, Sheela et al. used a SERS-DB-based workflow with 420 training and 180 test samples across six molecular classes; the reported classifier accuracy and F1-score were 92%, with Bayesian optimization used to refine sensor parameters [23]. These examples illustrate why independent or experimental spectra are a stronger test of the inference layer than random splits of a deterministic simulation grid.

Electrochemical biosensors provide another route to data-rich AI because voltammetric, amperometric and impedance measurements naturally generate multivariate features over voltage, frequency and time [4,24]. The important design principle is modality-agnostic: AI should be used where the raw measurement contains structure that fixed thresholds or one-dimensional calibration curves discard, while the mechanistic transducer model should remain visible enough to detect drift, confounding, and nonphysical extrapolation.

## 5. Explainable AI: From Feature Attribution to Physical Consistency

Explainable artificial intelligence (XAI) is particularly valuable in sensor engineering because many inputs have a physical interpretation. SHAP values can rank the contribution of wavelength, layer thickness, pitch, refractive index, or other design variables; LIME can provide local explanations around individual predictions. The foundational SHAP framework offers additive feature attributions [52], while LIME approximates a model locally with an interpretable surrogate [53]. In scientific applications, however, explanations should be treated as hypotheses about model behavior, not proof of a causal mechanism.

Dwivedi et al. applied both SHAP and LIME to a Gaussian-process model for fiber-optic SPR sensing and found metal thickness to be more influential than wavelength for FOM in the investigated domain, a result that they linked to known radiation-damping behavior [9]. This is a useful model of XAI practice: the explanation is checked against sensor physics. By contrast, a feature ranking that contradicts well-established physics may signal confounding, an overly narrow training domain, coding errors, or a model exploiting correlations created by the simulation design.

For biosensors, XAI should therefore be used in three layers. First, global explanations identify the variables that control performance across the design domain. Second, local explanations show why a particular design or sample received a prediction. Third, physical consistency checks compare the direction and magnitude of the learned dependence with known laws, simulations, or controlled experiments. This third layer is often missing but is essential if explainability is intended to support scientific understanding.

## 6. Physics-Informed AI for Biosensors

### 6.1. What “Physics-Informed” Should Mean

Physics-informed machine learning (PIML) has developed rapidly in scientific computing. Physics-informed neural networks (PINNs) incorporate differential-equation residuals and boundary or initial conditions into the learning objective [54], while broader PIML frameworks include neural operators, differentiable solvers, hybrid mechanistic/data-driven models and constrained learning [55]. The motivation is especially strong when data are scarce, extrapolation is required, or interpretability matters; these issues are increasingly recognized across biomedical science and engineering [56].

In biosensing, this distinction is operational rather than semantic. A regressor fitted to TMM or FEM outputs learns an interpolation map from a physics-generated dataset, but no physical residual is evaluated during optimization. It remains physics-guided even when its inputs and outputs are physically meaningful. A model becomes physics-informed, under the strict definition used here, only when violations of the governing model are penalized during training or excluded by construction.

For a neural field uθ(x) governed by a differential operator N and boundary operator B, a generic physics-informed objective can be written as L(θ) = λdata N_d^−1^∑_i_||uθ(x_i_) − y_i_||^2^ + λphys N_r^−1^∑_j_||N[uθ](x_j_) − f(x_j_)||^2^ + λbc N_b^−1^∑_k_||B[uθ](x_k_) − g(x_k_)||^2^. The first term fits measurements, the second evaluates the governing-equation residual at collocation points, and the third enforces boundary or initial conditions. The λ coefficients must be reported because they define the compromise between empirical fit and physical consistency.

For an optical SPR or THz problem, uθ may represent electric and magnetic fields, and N may contain the frequency-domain Maxwell residuals ∇ × Eθ − iωμHθ and ∇ × Hθ + iωεEθ − J. For electrochemical sensing, the same construction can combine Nernst–Planck transport, electroneutrality or Poisson electrostatics, and Butler–Volmer interfacial kinetics. For a GFET, a Poisson or Poisson–Boltzmann residual and boundary conditions at the electrolyte, oxide, and channel interfaces can constrain the learned potential and carrier-density fields. These are genuinely physics-informed formulations; merely using their numerical solutions as labels is not.

### 6.2. Hybrid Differentiable Models

A practical biosensor model need not be a large PINN. If a differentiable mechanistic model Fphys(c,ϕ) predicts the sensor response from analyte concentration c and physical parameters ϕ, a hybrid predictor can be written ŷ = Fphys(c,ϕ) + rθ(x), where rθ learns only structured discrepancy caused by roughness, heterogeneous binding, matrix effects, or drift. A concrete training objective is L(θ,ϕ) = N^−1^∑_i_||y_i_ − Fphys(c_i_,ϕ) − rθ(x_i_)||^2^ + λr||rθ||^2^ + λc∑_i_Pphys(ŷ_i_). Here Pphys can penalize negative concentrations, violation of known monotonic ranges, non-causal dynamics, or disagreement with independently measured material constants. Reporting each term and weight makes clear which constraints are embedded rather than applied after prediction.

For example, an electrochemical hybrid can place a neural discrepancy term on top of a Butler–Volmer/Nernst–Planck current model while preserving mass balance and electrode boundary conditions. An SPR hybrid can infer uncertain complex permittivity, effective binding-layer thickness, or refractive-index change through a differentiable TMM module and reserve the neural residual for systematic simulator-to-device mismatch. In both cases, the learned component is smaller and more auditable than an unconstrained end-to-end mapping.

Hybrid models also provide a natural route to inverse problems. Rather than predicting concentration from a spectrum with a generic network, the algorithm can infer the latent refractive-index or binding change that best explains the measured response through the physical forward model. Uncertainty on that latent quantity can then be propagated to the final concentration or diagnostic decision.

### 6.3. Uncertainty Is Part of the Prediction

A biosensor result without uncertainty is incomplete. Aleatoric uncertainty reflects irreducible or condition-dependent variability in measurement noise, sample matrix, and biological heterogeneity; epistemic uncertainty reflects limited training coverage, uncertain parameters, and model-form error. Gaussian-process regression provides a predictive distribution and is attractive for small design datasets, although its posterior uncertainty is only as credible as its kernel and noise model. Bayesian neural networks approximate a posterior over weights but require convergence and calibration checks; deep ensembles and Monte Carlo dropout provide practical approximations to epistemic variability, with ensembles generally offering a stronger empirical baseline at greater computational cost.

Uncertainty should also control system behavior. A device can be designed to abstain, request recalibration, or flag a measurement for confirmatory testing when the input lies outside the validated domain. This is more clinically responsible than forcing a confident class label from an unfamiliar sample.

Conformal prediction addresses a different objective: using a held-out calibration set, it converts residuals or class scores into prediction intervals or sets with finite-sample marginal coverage under exchangeability. It does not identify the physical source of uncertainty, and its guarantee may fail after device, matrix, site, or population shift. For biosensors, calibration data should therefore be grouped at the intended deployment unit—for example by device, fabrication batch, patient, or site—and coverage should be reported both overall and within relevant matrix or subgroup strata.

When an uncalibrated biological matrix or an out-of-distribution sample is encountered, GPR variance, ensemble disagreement, or Bayesian posterior spread may rise, but none is guaranteed to detect every shift. A deployable workflow should combine predictive intervals with an explicit OOD score, negative and interference controls, and a prespecified action such as abstention, recalibration, dilution, or confirmatory testing. UQ should be evaluated with interval coverage and width for regression, calibration and selective-risk curves for classification, and stress tests across held-out devices, batches, matrices, and sites.

## 7. Representative Evidence Across the Validation Ladder

### 7.1. In Silico Optimization and Surrogates

Simulation-led studies show where ML adds engineering value without establishing analytical or clinical performance. Across PCF-SPR, fiber-optic SPR, and multilayer 2D-material platforms, regressors accelerate FEM/TMM evaluation, SHAP or LIME identifies influential geometric variables, and Monte Carlo perturbations expose fabrication-sensitive optima [8,9,10,49,50]. Under the terminology adopted here, these are physics-guided surrogates because governing equations generate the training labels but are not enforced by the fitted regressors.

The cross-cutting trend is a shift from maximizing one nominal sensitivity value toward robust multi-objective design. Useful outputs are therefore Pareto sets that jointly consider sensitivity, linewidth or FOM, optical loss, tolerance to thickness and material uncertainty, and distance from the training-domain boundary. The dominant error profile at this level is interpolation leakage from densely sampled numerical grids, followed by extrapolation and simulator-to-device mismatch.

Evaluation should hold out contiguous parameter ranges or complete material/geometry combinations, not only random grid points. The next validation step is fabrication of multiple independently produced devices and estimation of the residual discrepancy between measured and simulated responses. Table 2 orders representative studies by the evidence hierarchy so that simulator surrogates are not ranked directly against clinical classifiers.

### 7.2. Device-Level Experimental Validation

At device level, AI operates on measured responses and must absorb nuisance variation absent from deterministic grids. Graphene chemosensor arrays, PCB-integrated GFETs, electrolyte-gated GFETs, and wearable electrochemical platforms use classification or regression to combine multichannel signals, compensate device variability, or estimate analyte concentration [11,12,13,14,21]. Their shared challenge is transfer across independently fabricated devices, batches, operators, and time points; a random split of repeated measurements from the same device does not test that claim.

Fabricated THz and electrochemical platforms provide complementary device-level tests in realistic media. Metal–graphene THz sensing has been demonstrated with circulating tumor DNA in patient plasma [15], and all-dielectric THz devices provide measured glucose benchmarks in controlled solutions and human serum [19,20]. These results are more informative than simulator accuracy for hardware closure, but they still require blinded replication, batch-aware validation, and reference-method agreement before clinical inference can be claimed.

Across modalities, the dominant device-level errors are calibration drift, substrate or hot-spot variability, non-specific adsorption, matrix interference, and between-device offsets. Models should therefore be evaluated with leave-one-device- or leave-one-batch-out splits, repeated calibrations over time, interference panels, and explicit failure criteria. These issues are illustrated by the interpretable simulation design space and measured GFET inference workflow discussed below.

### 7.3. Cohort-Level Clinical and Matrix Validation

Cohort-level studies change the validation unit from a spectrum or device reading to an independent participant or specimen. SERS studies using patient-derived serum illustrate this transition, including two-cohort pan-cancer classification (1582 cancer cases and 382 controls) [61] and a large case-control early-cancer study (1655 cases and 1896 controls) [62]. Electrochemical classification using 531 urine samples provides a complementary clinical-sample example [16]. These sample counts increase statistical power, but retrospective case-control performance still does not establish prospective predictive value at deployment prevalence.

The main errors at this level are patient leakage, spectrum-level rather than participant-level splitting, demographic or site confounding, prevalence shift, and batch effects correlated with disease labels. Validation should therefore preserve participant independence, report cohort construction and disease stage, separate model selection from final testing, and—where possible—use an external site or prospective cohort. The evidence comparison below displays reported participant/sample counts for the cohort-level examples and labels unavailable counts as not reported (NR).

Figure 3 and Figure 4 serve different purposes. Figure 3 illustrates within-modality variability but cannot remove architecture dependence. A useful dimensionless optical comparison is the fractional refractive-index resolution Rn = (δnmin/n0), where δnmin is the smallest resolvable refractive-index change, and n0 is the baseline index. When δnmin is not directly reported, an instrument-aware estimate is δnmin≈δqmin/|∂q/∂n|, where q is angle, wavelength, frequency, or another measured resonance coordinate and δqmin is the experimentally resolvable shift. For concentration sensing, a normalized limit of detection can be stated as LoD/cmax (or relative to a clinically defined threshold), but only when matrix, analyte, calibration range, and confidence rule are identical enough to make the ratio meaningful.

These normalized quantities improve reporting but do not make optical, electrical, and electrochemical platforms universally interchangeable. Electrical and electrochemical studies should additionally report signal-to-noise or baseline-normalized response, dynamic range, recovery, and reference-method agreement. Accordingly, cross-modality benchmarking in Table 2 is organized primarily by data provenance, validation unit, and evidence level, while modality-specific performance remains in its native units.

The link between physically meaningful design variables and sensor performance is illustrated more directly in Figure 5. In the graphene SPR study of Tene et al. [46], the number of graphene layers and the metallic-layer sequence produced systematic changes in sensitivity enhancement. This type of response surface is well suited to physics-guided optimization because the input variables retain a clear material and optical interpretation.

An experimental counterpart is shown in Figure 6. Zhang et al. [21] combined aptamer-functionalized electrolyte-gated graphene field-effect transistor measurements with regression models that use measured electrical features to estimate Ca^2+^ concentration. Here, machine learning acts directly on experimental transduction data rather than on a simulated parameter sweep, providing a useful example of how device physics and data-driven inference can be integrated.

## 8. Benchmarking: What Should Be Reported?

AI-enabled biosensor papers often report model metrics more completely than sensor validity. A regression table containing R^2^, MAE, and RMSE is useful, but it does not answer whether a model is physically consistent, robust to fabrication error, or transferable to independent devices. The evaluation unit must match the intended application. If the claim is “works on a new fabricated sensor,” the test set must contain sensors that were not represented in training. If the claim is “works across patients,” the test set must contain patients that were not represented in training.

For simulation-trained models, the sampling strategy should also be disclosed. Regular parameter grids can create strongly autocorrelated neighboring points; random train/test splitting then makes prediction easier than true design-space interpolation. More demanding evaluation can hold out entire thickness ranges, material combinations, or refractive-index regimes and test whether the surrogate fails gracefully outside its training envelope. The corresponding minimum reporting requirements are summarized in Table 3.

A useful minimum classification is therefore: Level A, material or device physics only; Level B, full-wave or TMM device simulation; Level C, simulation-trained ML with held-out numerical domains; Level D, fabricated device with measured spectra; Level E, analyte calibration in realistic matrices; and Level F, external or prospective biomedical validation. Table 2 uses these levels to prevent direct comparison of fundamentally different evidence types. A model should not move upward in the hierarchy merely because its R^2^ increases.

## 9. From Simulation to Fabrication and Biological Samples

The largest gap in AI-assisted plasmonic biosensor papers is often not algorithmic but experimental. Simulations typically assume nominal thicknesses, tabulated optical constants, uniform layers, fixed temperature, and ideal analyte refractive indices. Fabricated devices introduce roughness, grain structure, adhesion layers, oxidation, contamination, and thickness deviations. Biological samples introduce non-specific adsorption, viscosity changes, competing species, variable ionic strength, and uncertain receptor activity.

Accordingly, the first experimental use of AI should often be calibration of the simulation-to-device discrepancy. A small number of fabricated sensors can be used to estimate systematic offsets or learn a residual correction on top of the physical model. Active learning can then select the next most informative designs to fabricate, reducing experimental cost. This closes the loop between modeling and experiment and is more data-efficient than training a large network from scratch.

For 2D materials, long-term stability deserves special attention. BP degradation, graphene transfer defects and interface contamination can change the effective optical or electrical properties over time. A model that uses device age, environmental history, and calibration controls as inputs may be more useful than one that assumes a static material. However, such a model must be validated prospectively to avoid learning site-specific maintenance patterns rather than sensor physics. The main stages required to move from simulated performance to biomedical validation are summarized in Figure 7.

Independent experiments show how the standard of evidence changes as validation moves from simulation to measured devices and biological samples. The LSPR/ML SARS-CoV-2 platform of Liang et al. linked optical images to direct particle detection rather than to a refractive-index proxy [22]. More recent graphene, GFET, wearable, and SERS studies extend this principle to cross-device chemical sensing, multianalyte transistor detection, on-body sweat measurements, and patient-derived serum spectra [11,12,13,14,61,62,63,64,65,66,67,68,69,70]. The scale of the evidence ranges from individual fabricated devices to thousands of clinical samples. Particularly informative examples include independent cohorts [61], patient plasma measured with fabricated metal–graphene THz hardware [15], and hundreds of urine samples analyzed with an electrochemical immunosensor and ML [16]. Together, these studies define the type of external evidence that future graphene/THz AI biosensors should seek.

Recent reviews reach similar conclusions. Across SPR [2], electrochemical sensing [28,29,30,45], point-of-care biosensors [31], microfluidics [32], SERS [33,34,35,36,37,38,43], FET platforms [41,42], graphene THz devices [44] and broad multimodal biosensing [39,40], the recurring needs are richer information-bearing measurements, transparent dataset construction, device/batch-aware validation, calibration transfer and deployment-oriented uncertainty. The main challenge is no longer a lack of possible algorithms; it is showing that performance survives changes in hardware, matrix, operator, site, and population while remaining tied to the measurement physics. Representative experimental and clinical studies are compared in the consolidated Table 2.

Table 2 also clarifies why ‘experimental’ is not a single category. A fabricated resonator measured in buffer (Level D/E) answers a different question from a classifier evaluated on hundreds or thousands of patient samples (Level F). Conversely, a large clinical dataset cannot compensate for unstable sensor physics if substrate, batch, or preprocessing effects are uncontrolled. Future studies should therefore combine hardware reproducibility with cohort-level external validity.

## 10. Trustworthy AI and Clinical Translation

Clinical translation changes the standard of evidence. A sensor model becomes part of a measurement system whose errors can affect diagnostic or therapeutic decisions. High model performance on a development dataset is therefore only one component of trustworthiness. Transparency, uncertainty, human factors, subgroup performance, cybersecurity, change control, and post-deployment monitoring become relevant, consistent with broader analyses of the obstacles to clinical impact from AI [71].

Rudin has argued that high-stakes decisions should favor inherently interpretable models when feasible rather than relying exclusively on post hoc explanations of black boxes [72]. This principle fits biosensing well: many problems can be solved with compact regressors, calibrated probabilistic models, or hybrid physical models whose parameters are understandable. Deep architectures are justified when signal structure demands them, but complexity should solve a demonstrable problem rather than serve as a proxy for novelty.

International regulatory discussions increasingly emphasize lifecycle thinking for ML-enabled medical devices. Joint FDA/Health Canada/MHRA transparency principles highlight communication of intended use, inputs, outputs, performance, limitations, data characteristics, logic or explainability when available, and ongoing monitoring [73]. Good Machine Learning Practice principles similarly emphasize representative datasets, separation of training and test sets, clinically relevant testing, human–AI team performance, and monitoring of deployed models [74]. For intelligent biosensors, these principles imply that the AI layer cannot be documented independently of the physical device and its use environment.

## 11. Research Agenda: Toward Autonomous but Auditable Biosensors

- Physics-constrained surrogate models that preserve the speed of ML while enforcing electromagnetic, electrochemical, or kinetic consistency.
- Multi-objective inverse design that includes sensitivity, FOM, selectivity, stability, fabrication tolerance, and cost rather than maximizing one idealized metric.
- Uncertainty-aware prediction with explicit out-of-distribution detection and abstention rules.
- Active-learning loops in which the model selects the next simulation or fabrication experiment that maximally reduces uncertainty.
- Device- and batch-aware validation to quantify generalization across fabrication runs and laboratories.
- Cross-modal sensing that combines optical, electrochemical, and environmental channels while retaining interpretable physical submodels.
- Digital twins of biosensors that update material/interface parameters during use and separate reversible calibration drift from irreversible device degradation.
- Prospective validation in realistic biological matrices, followed by external and clinical workflow studies when a medical claim is intended.

The most promising endpoint is not a completely opaque “autonomous sensor.” It is an auditable system that can redesign or recalibrate itself within predefined physical and regulatory boundaries, estimate its own uncertainty, expose the main reasons for a prediction, and recognize when it is operating outside its validated domain. Such systems would make AI a component of measurement science rather than an add-on to signal processing.

## 12. Conclusions

Nanomaterial-based biosensors offer a strong opportunity for responsible AI because their data-driven tasks are anchored in identifiable physical transduction mechanisms. Experimental nanocarbon electrochemistry [5], graphene/TMD SPR parameter studies [3,46,47,48], and independent ML/XAI-assisted optical modeling [8,9,10,49,50] illustrate the progression from material-level transduction to data-driven design. The broader 2023–2026 literature increasingly moves beyond simulator surrogates: graphene and GFET platforms address device variability [11,12,21,75], wearable electrochemistry produces user-facing time-series data [13,14], SERS/ML studies operate on substantial clinical serum cohorts [61,62,63,64,65,66,67,68,69,70], and fabricated THz and electrochemical systems increasingly incorporate realistic biological matrices [15,16,17,18,19,20]. These developments support evaluating AI by evidence quality and generalization as well as by predictive accuracy.

The next generation of intelligent biosensors should combine mechanistic models with data-driven residual learning, uncertainty quantification, explainability validated against known physics, fabrication-tolerance analysis, and genuinely independent experimental datasets. For graphene, black phosphorus, and related low-dimensional materials, this approach is particularly valuable because the performance benefits of the nanomaterial are coupled to nontrivial trade-offs in optical loss, stability, surface chemistry, and integration quality. Physics-informed and explainable AI can turn these trade-offs from a parametric burden into an auditable design strategy.

Ultimately, the decisive metric will not be whether an algorithm predicts simulated sensitivity with R^2^ > 0.99. It will be whether the complete biosensor system remains accurate, calibrated, interpretable, and safe when material batches change, devices age, real biological matrices are introduced, and decisions are made outside the laboratory.

## Figures and Tables

**Figure 1:**
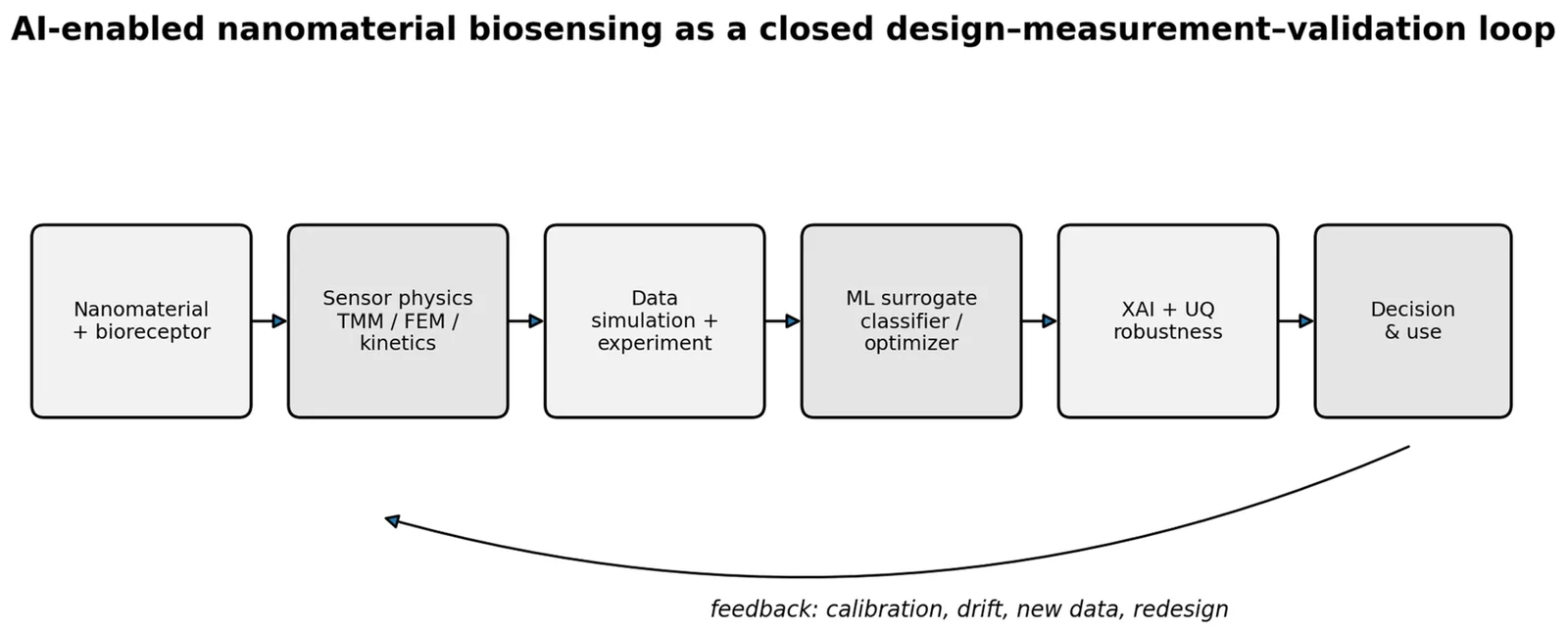
Closed-loop view of AI-enabled nanomaterial biosensing. AI can act before measurement (design and simulation), during measurement (reconstruction and calibration), and after measurement (inference and decision support). The central requirement is feedback between data-driven models and sensor physics. Abbreviations: AI, artificial intelligence; ML, machine learning; XAI, explainable artificial intelligence; TMM, transfer-matrix method; FEM, finite-element method.

**Figure 2:**
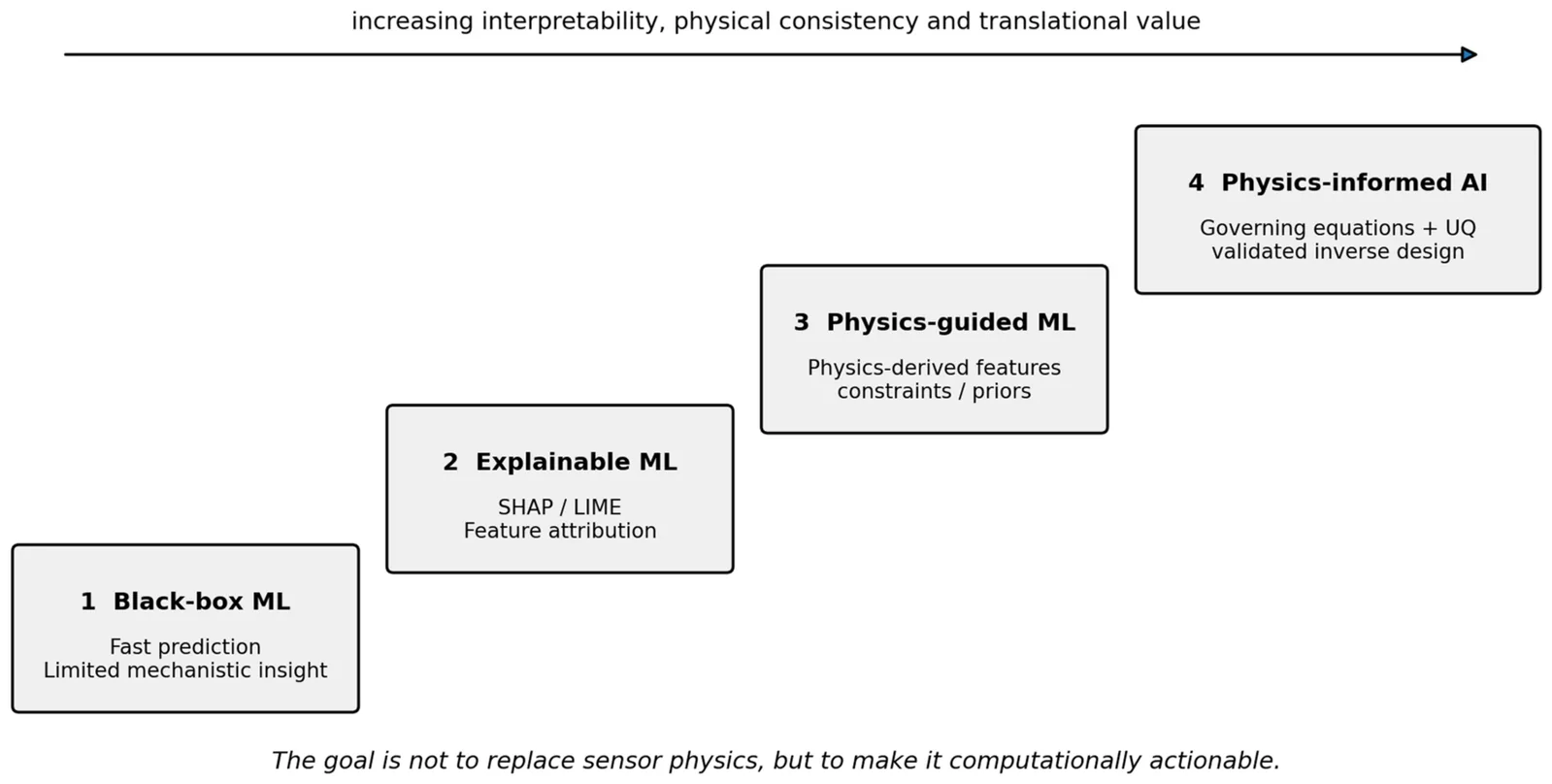
Stages of AI integration in biosensor engineering. Explainability and physics constraints add information beyond predictive performance but do not replace experimental validation.

**Figure 3:**
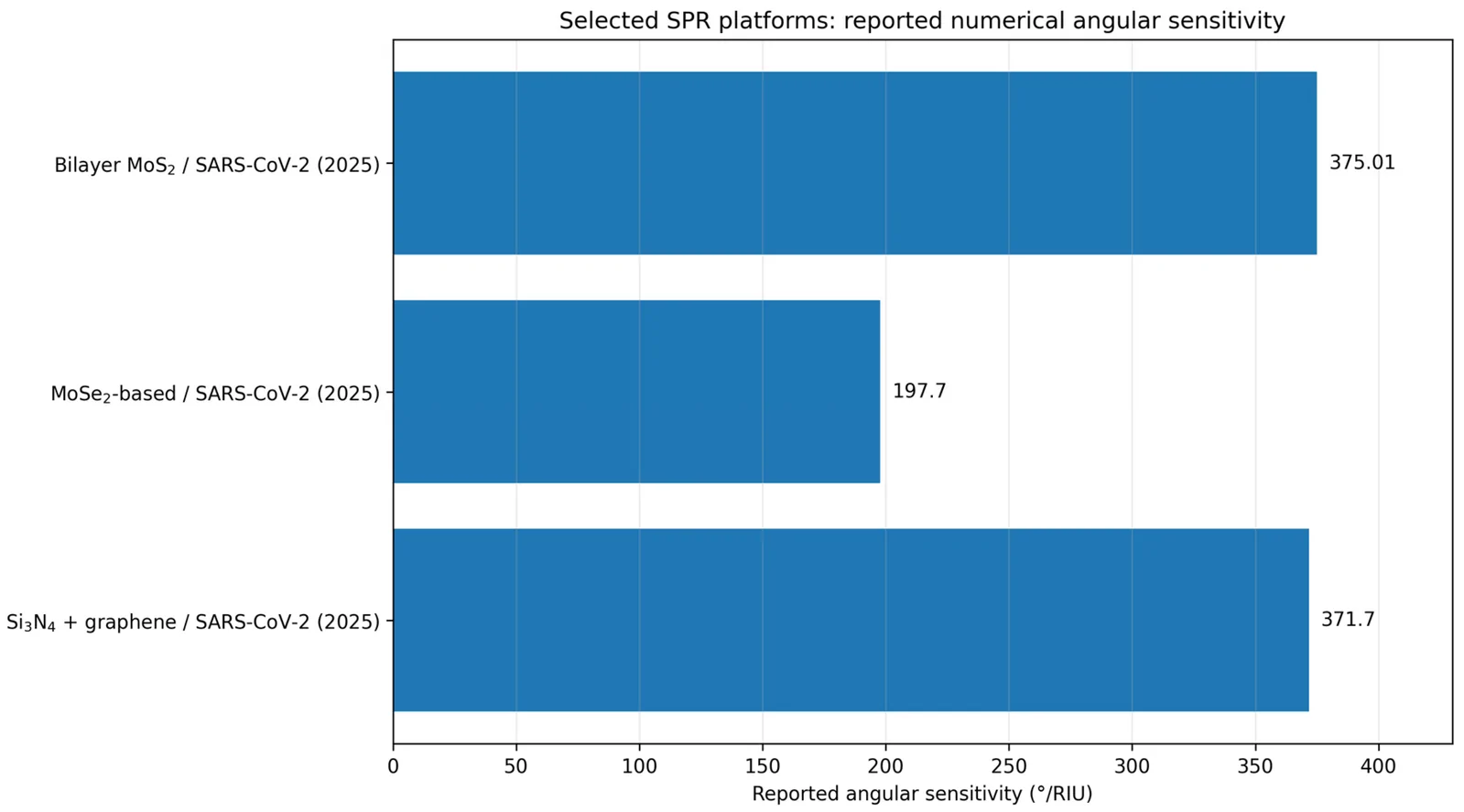
Comparison of reported angular SPR sensitivity in selected numerical studies [3,47,48]. Values are shown in the source units (°/RIU) and are not a scalar ranking: they must be interpreted with operating wavelength, refractive-index interval, metal/dielectric stack, linewidth, figure of merit, optical loss, and evidence level. The plot was created by the author from reported numerical values; no published artwork was reproduced.

**Figure 4:**
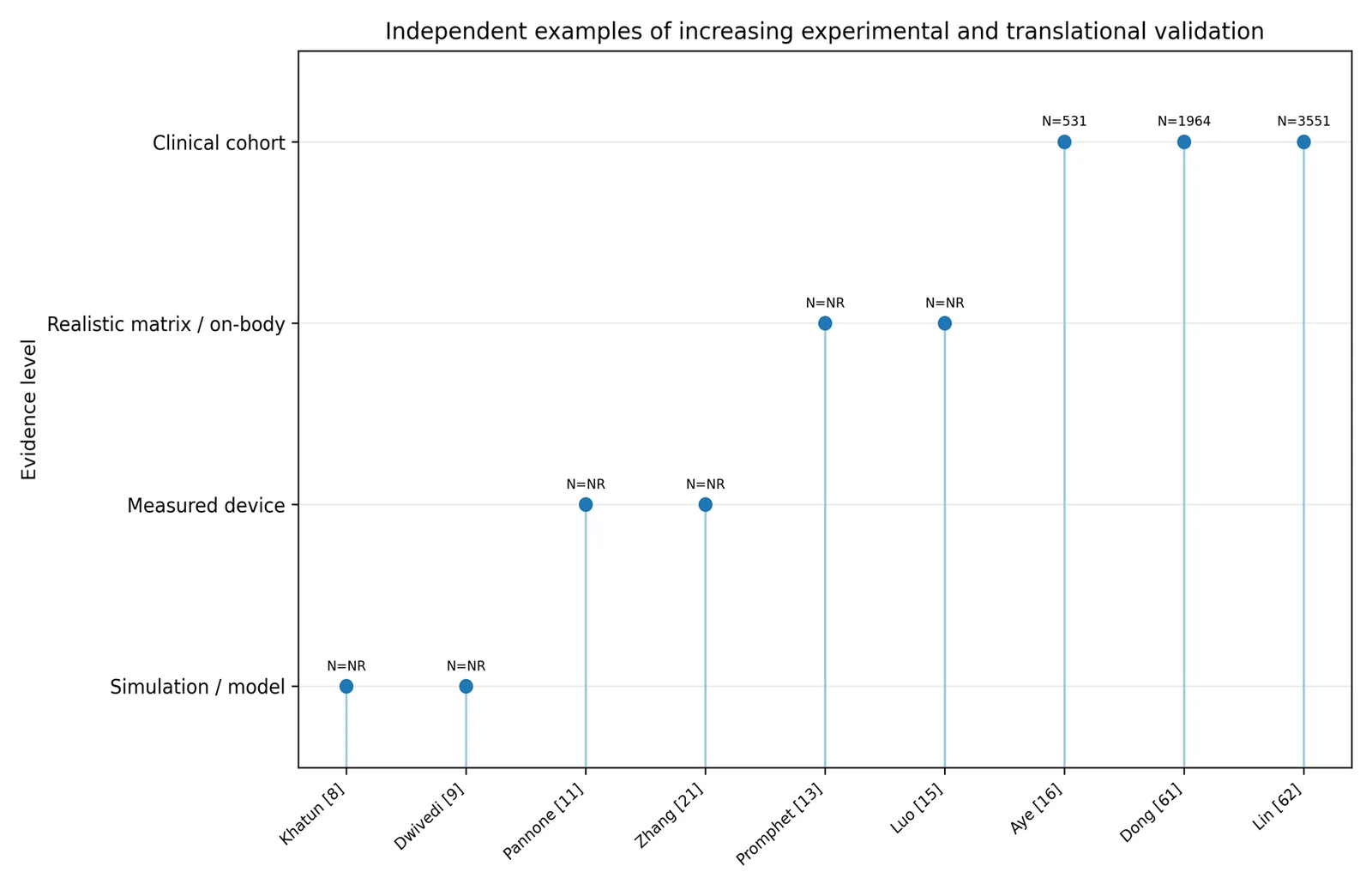
Evidence maturity across selected independent AI-enabled biosensing studies [8,9,11,13,15,16,21,61,62]. Labels show reported participant/sample counts for cohort-level studies (N = 531, 1964, and 3551, respectively); NR indicates that a comparable sample/device count was not reported in the review dataset. Evidence levels distinguish simulation/model studies, measured-device studies, realistic-matrix or on-body measurements, and clinical cohorts. The blue points and guide lines locate individual studies on these evidence categories; they do not represent measured continuous trajectories.

**Figure 5:**
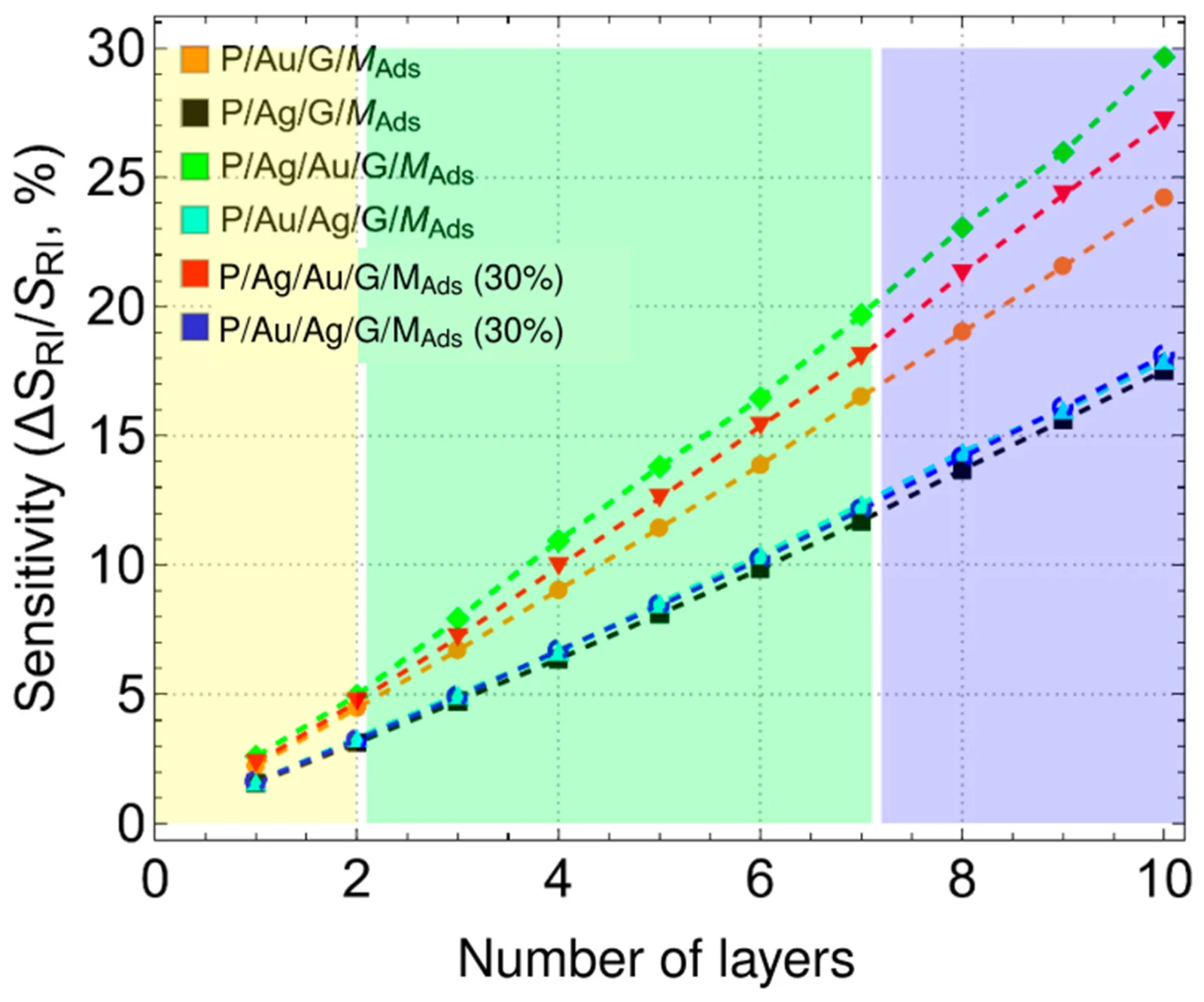
Sensitivity enhancement relative to a conventional SPR biosensor as a function of graphene layer number for different metallic-layer configurations. The plot illustrates a physically structured design space in which material choices remain directly interpretable. Colored background bands distinguish low, intermediate, and higher graphene-layer counts; the band colors do not represent separate measurements. Adapted from Ref. [46], originally published by MDPI in Sensors. © 2024 by the authors, licensed under the Creative Commons Attribution 4.0 International License (CC BY 4.0).

**Figure 6:**
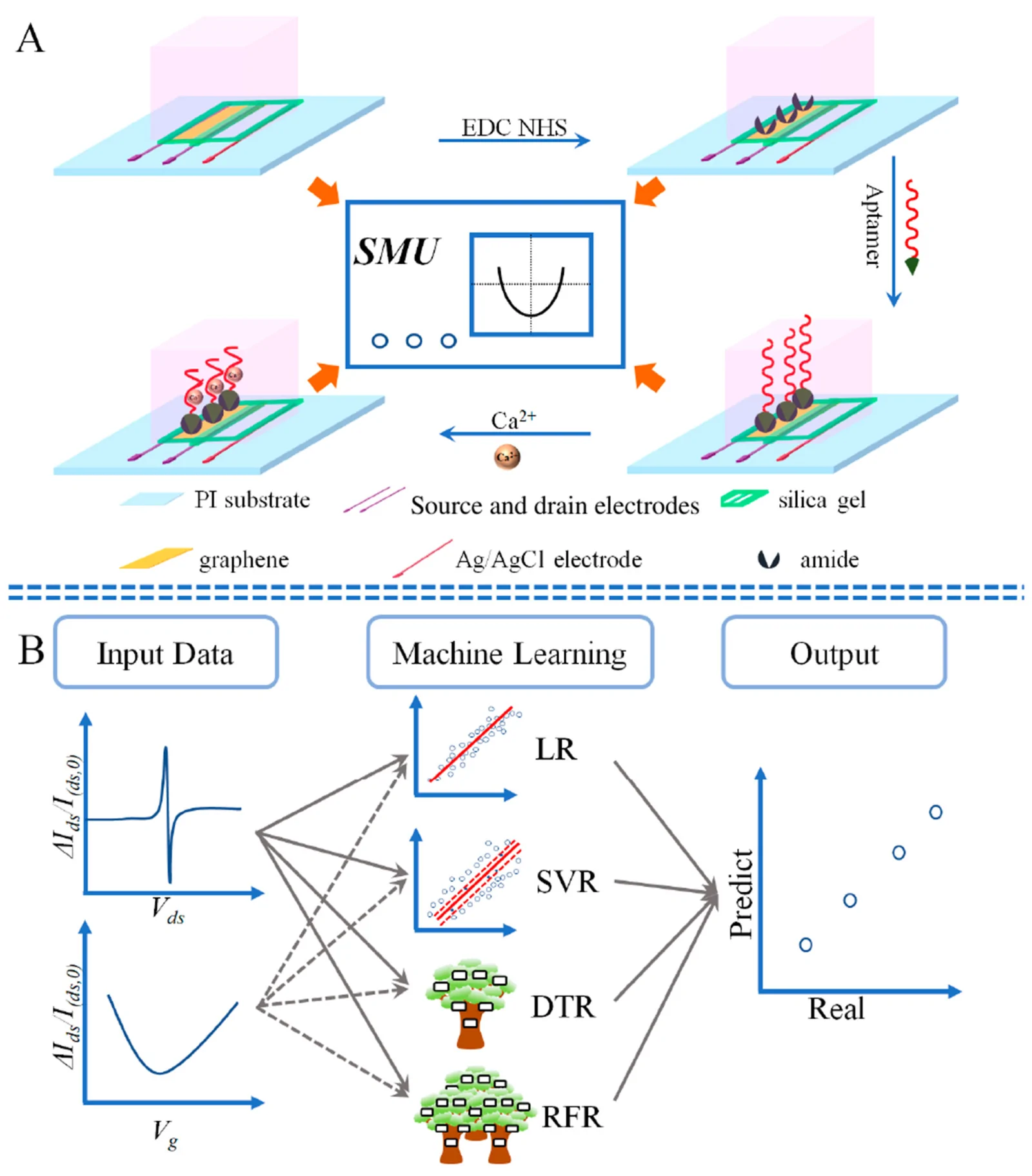
Experimental and machine-learning workflow for calibration-free Ca^2+^ determination with an electrolyte-gated graphene field-effect transistor. (**A**) Sensor functionalization with a Ca^2+^-sensitive aptamer and electrical measurement; (**B**) measured electronic features processed by linear regression (LR), support vector regression (SVR), decision-tree regression (DTR), and random-forest regression (RFR) to predict Ca^2+^ concentration. Solid and dashed arrows indicate feature paths to candidate regression models; colored layers in panel A distinguish the illustrated substrate, graphene, electrodes, silica gel, and aptamer functionalization. Adapted from Ref. [21], originally published by MDPI in Sensors. © 2022 by the authors, licensed under the Creative Commons Attribution 4.0 International License (CC BY 4.0).

**Figure 7:**
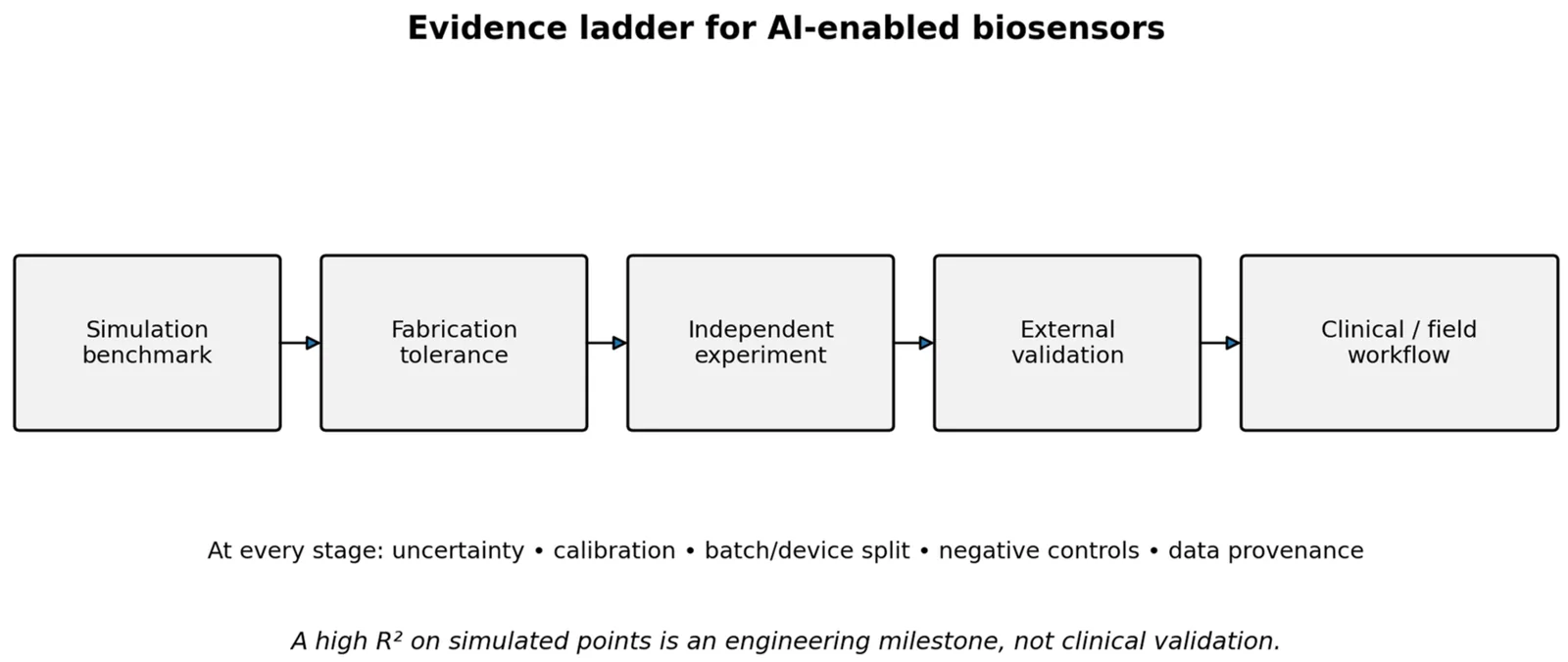
Validation pathway from simulation to biomedical use. Progression toward practical application requires fabrication-tolerance analysis, experimental reproducibility, external validation, and workflow-level assessment.

**Table 1:** Main AI functions across the nanomaterial-biosensor lifecycle. Abbreviations: AI, artificial intelligence; ML, machine learning; RF, random forest; XGBoost, extreme gradient boosting; GPR, Gaussian process regression; ANN, artificial neural network; CNN, convolutional neural network; SVM, support vector machine; S, sensitivity; FOM, figure of merit; FWHM, full width at half maximum.

Stage	Typical Inputs	AI Task	Typical Models	Useful Outputs	Main Failure Mode
Material/device design	materials, thicknesses, geometry	forward surrogate; multi-objective optimization	RF, XGBoost, GPR, ANN	S, FOM, FWHM, loss	extrapolation outside simulated domain
Inverse design	desired response/performance	recover feasible design variables	surrogate + optimizer; generative models	candidate stacks/geometries	non-unique or non-fabricable solutions
Signal processing	spectra, voltammograms, impedance, time series	denoising; feature extraction	CNN, autoencoder, filters + ML	clean signal; compact features	removal of weak true signal
Analyte inference	features + metadata	regression/classification	SVM, RF, boosting, neural networks	concentration, class, probability	data leakage; matrix confounding
Calibration/drift	reference signals, temperature, device history	domain adaptation; recalibration	online ML, transfer learning	stable response over time	silent performance decay
Decision support	sensor output + uncertainty	risk/decision model	calibrated classifiers, Bayesian models	interpretable recommendation	overconfidence; poor external validity

**Table 2:** Consolidated evidence matrix for representative AI-enabled nanomaterial and biosensor studies, sorted by evidence level. NR, not reported in the review dataset; levels A–F are defined in Section 8.

Evidence	Study	Platform/Material	Data Provenance/N	AI Role	Validation Unit	Key Result/Contribution	Main Limitation
A	Tene et al. 2024 [46]	Graphene multilayer SPR	TMM sweep	Parametric optimization	Numerical design	Maps layer/stack trade-offs	No ML or experimental validation
C	Dwivedi et al. 2024 [9]	Fiber-optic SPR	Modelled design domain; N NR	GPR + SHAP/LIME	Held-out numerical points	Physics-checked feature attribution	Limited domain; grid leakage risk
C	Khatun & Islam 2025/2026 [8,49]	PCF-SPR	FEM grids; N NR	Multiple regressors + SHAP/UQ	Held-out numerical points	Fast surrogate, attribution and UQ	No fabricated-device validation
C	Patel et al. 2024 [10]	2D-material SPR	Numerical SPR dataset	ML optimization	Numerical designs	Optimizes multilayer architecture	Predominantly numerical evidence
C	Anik & Imam 2026 [50]	Multilayer SPR arsenic sensor	TMM + FEM + Monte Carlo	Regression + SHAP	Held-out simulations/perturbations	Adds tolerance analysis	Experimental closure absent
C	Ponlatha et al. 2026 [57]	BP–SrTiO3 SPR glucose sensor	TMM/Maxwell numerical dataset; N NR	ML-assisted optimization	Held-out numerical designs	Extends surrogate design to BP heterostructures	Fabrication and matrix validation absent
D	Pannone et al. 2024 [11]	Graphene chemosensor array	Measured devices/conditions; N NR	Supervised ML	Across devices and conditions	Targets cross-device robustness	Clinical matrix/site transfer absent
D/E	Mukherjee et al. 2025 [12]	PCB-integrated GFET	Measured antibiotic responses; N NR	XGBoost classification/regression	Experimental chip responses	Parallel antibiotic detection	Long-term drift/site validation needed
E	Promphet et al. 2024 [13]	PB/CNT-CNF wearable	On-body sweat; N NR	Regression/optimization	Wearable measurements	Integrated smartphone glucose sensing	Larger prospective cohorts needed
E	Zhou et al. 2024 [14]	Wearable electrochemical interface	Human multichannel sweat; N NR	Multivariate regression	Persons/sessions	Explainable biochemical prediction	Inter-person calibration transfer
E	Luo et al. 2024 [15]	Metal–graphene THz metasurface	Fabricated device + patient plasma; N NR	Signal-analysis workflow	Device + clinical matrix	Sub-fM ctDNA; SNP selectivity	Larger blinded cohorts needed
D/E	Zhang et al. 2023 [21]	Electrolyte-gated GFET	Measured transfer/output curves; N NR	LR/SVR/DTR/RFR	Measured device	Calibration-free Ca^2+^ estimation	Batch and matrix transfer needed
D/E	Reddy et al. 2023 [58]	AuNP-decorated CNT-FET	Measured biosensor responses; N NR	Data-assisted inference	Measured device	Broadens experimental FET evidence	Independent batch/site validation needed
D/E	Gubeljak et al. 2023 [59]	Electrochemically gated graphene microwave sensor	Measured microwave response; N NR	Signal interpretation	Measured device	Links graphene transduction to microwave readout	Broader matrix and external validation needed
E	Liu et al. 2026 [19]	All-dielectric THz	Fabricated glucose measurements; N NR	No ML; benchmark	Measured device	Measured high-Q reference	Not cohort-level validation
E	Shi et al. 2026 [20]	Functionalized THz	Human-serum glucose; N NR	Quantitative spectral analysis	Serum + reference comparison	Realistic-matrix hardware closure	Broader patient/site validation
E	Dai et al. 2023 [60]	Metal–graphene THz metasensor	Measured pesticide response; N NR	Signal-analysis workflow	Fabricated device	Experimental metal–graphene THz benchmark	Clinical/biological validation not applicable
F	Dong et al. 2023 [61]	Ag-nanowire SERS-AICS	1582 cancer + 382 controls; N = 1964	ML classification	Two cohorts/participants	95.81% pan-cancer accuracy	Retrospective; prospective workflow needed
F	Lin et al. 2025 [62]	Serum SERS + deep learning	1655 cases + 1896 controls; N = 3551	Deep classification	Participants	Large early-cancer case-control study	Prevalence and prospective effects
F	Aye et al. 2026 [16]	Electrochemical immunosensor	Urine samples; N = 531	Six classifiers	Participant-level CV	90.65% best accuracy	External geographic validation needed

**Table 3:** Recommended minimum reporting checklist for AI-enabled nanomaterial biosensors.

Domain	Minimum Item	Why It Matters	Preferred Evidence
Dataset	Source, sample/device counts, parameter ranges, missing data, preprocessing	Defines what the model has actually seen	Machine-readable data + provenance
Split strategy	Prespecified group-based split by sample, patient, device, batch, site, or simulation trajectory; all technical replicates kept within one fold	Prevents replicate, neighbor-grid, device, and patient leakage that inflates performance	GroupKFold/leave-one-group-out; nested tuning; untouched external group
Cohort structure	Inclusion/exclusion criteria, prevalence, disease stage, demographics when relevant	Determines clinical transportability	Independent cohort or prospective recruitment
Physics	Governing model, constants, boundary conditions, numerical convergence	Allows physical audit and reproduction	TMM/FEM/kinetic cross-check
Material realism	Measured optical/electrical constants, thickness spread, surface chemistry, aging	Constrains simulator-to-device mismatch	Batch-specific metrology and tolerance distributions
ML	Model, hyperparameters, tuning protocol, and baselines	Separates true gain from complexity	Nested CV or untouched test set
XAI	Global + local explanation and physical consistency check	Makes attribution scientifically useful	SHAP/LIME or interpretable model compared with known trends
Uncertainty	Prediction intervals, calibration and out-of-distribution behavior	Identifies low-confidence outputs	Coverage/calibration curves + abstention policy
Robustness	Fabrication, noise, temperature, matrix, interferents, and drift perturbations	Tests realistic operating conditions	Monte Carlo + experimental perturbation
Batch/device transfer	Performance across fabrication lots and individual sensors	Tests whether the model learned one device	Leave-one-device/batch-out evaluation
Calibration transfer	Recalibration burden across instruments, substrates and sites	Critical for spectroscopy and wearable deployment	Predefined transfer protocol; no test-set refitting
Experiment	Independent fabricated devices and biological samples	Bridges simulation to sensing	Blinded or external validation
Reference method	Agreement with accepted analytical/clinical comparator	Converts sensor output into meaningful evidence	Bland–Altman, recovery, reference-lab comparison
Clinical use	Intended-use population, decision threshold, failure handling	Connects model output to patient-facing claim	Prospective workflow validation
Reproducibility	Code/model version, data availability, random seeds, software stack	Allows audit and independent replication	Open code/data or controlled-access reproducibility package

## Data Availability

No new primary experimental data were generated. Values summarized or replotted in this review were taken from the cited published studies.
